# Foxp3+ Regulatory T-cells and IL-2: The Moirai of T-cell Fates?

**DOI:** 10.3389/fimmu.2012.00179

**Published:** 2012-07-12

**Authors:** Georg Gasteiger, Wolfgang Kastenmuller

**Affiliations:** ^1^Immunology Program, Memorial Sloan-Kettering Cancer CenterNew York, NY, USA; ^2^The Laboratory of Systems Biology, National Institute of Allergy and Infectious Diseases, National Institutes of HealthBethesda, MD, USA

Regulatory T-cells (T_reg_) have emerged as a crucial cellular checkpoint acting to prevent potentially harmful immune responses. Due to its highly diverse activities, regulating the immune system requires more than general suppression. Particularly, during the developing immune response to infection, T_reg_ need to balance the host reaction to achieve both an effective response against the invading pathogen as well as to prevent immunopathology from excessive or inappropriate activity. In this opinion article we discuss the dual roles of IL-2 as both a key inducer of T_reg_ activity and also a target of T_reg_ control during the acute phase of infection. We propose a model in which Foxp3+ regulatory T-cells dynamically “measure” IL-2 availability and restrict its access to effector T-cells, thereby controlling differentiation of these useful but potentially harmful cells.

CD4+ T-lymphocytes play a central role in orchestrating immune responses by modifying the functionality of other immune cells and guiding the qualitative features of a response to one optimal for resisting a particular microorganism. Besides augmenting both innate and adaptive immune responses, CD4+ T-cells limit excessive immune activation and immunopathology during infections. Among CD4+ T-cells, Foxp3+ regulatory T-cells (T_reg_) are essential for the maintenance of peripheral tolerance (Kim et al., 2007; Lahl et al., 2007). These cells also modulate the responses to pathogens (Belkaid and Tarbell, 2009). A plethora of mechanisms for how T_reg_ exert their function have been suggested (Shevach, 2009; Josefowicz et al., 2012). However, there is still an ongoing debate as to which functions of T_reg_ are essential under which circumstances. Likely, T_reg_ function can be attuned to specific conditions and distinct “rules” govern T_reg_ behavior in the steady-state versus inflammatory environments, secondary lymphoid (SLO) versus peripheral organs, developing versus ongoing immune responses, or acute versus chronic infections, for example. In this article we focus on the function of T_reg_ in the SLO during a developing acute infection and, although several cytokines are relevant, we concentrate on IL-2 as a central platform that enables effective immune control, as it (1) links activation of effector and regulatory responses, (2) establishes a feed-back loop for T-cell expansion, and (3) allows control over T-cell differentiation and fate decisions, preserving memory formation.

## IL-2 is a Central Cytokine for T-Cell Activation

IL-2, originally discovered as a mitogenic factor for T-cells, is bound as a quaternary complex with CD25 (IL2Rα-chain), CD122 (IL2Rβ-chain), and CD132 (common γ-chain). The α/β-heterodimer facilitates IL-2 capture with high affinity and, further stabilized by the γ-chain, forms a very stable complex which is terminated via receptor internalization rather than ligand dissociation (Smith, 2006). Upon activation by TCR interactions and additional co-stimulation via CD80/CD86, conventional T-cells (T_conv_) produce IL-2 and upregulate CD25 expression, which enhances IL-2 capture and consequently IL-2 signaling, further promoting CD25 expression, T-cell activation, and proliferation. This feed-forward loop can lead to activation-induced cell death, but highly activated, proliferating T-cells also undergo apoptosis when acutely deprived of IL-2 signals. Therefore, IL-2 is a master regulator of T-cell activation, proliferation, and death, excellently reviewed in Malek and Castro (2010), Boyman and Sprent (2012).

## Activation of T_reg_ Through IL-2: Anticipation and Sensing of Effector Responses

In contrast to conventional T-cells (T_conv_), T_reg_ constitutively express CD25 (Sakaguchi et al., 1995) and have STAT5 phosphorylation in the steady-state, arguing for continuous or high frequency intermittent IL-2 signaling in the absence of infection. Indeed, IL-2 signals seem to be pivotal for T_reg_ survival because animals that lack IL-2, CD25, or CD122 are largely devoid of peripheral T_reg_ and suffer from severe autoimmunity (Sadlack et al., 1993; Suzuki et al., 1995; Willerford et al., 1995; Fontenot et al., 2005). T_reg_ do not produce IL-2 themselves when stimulated through the TCR and therefore rely on paracrine IL-2 for their maintenance. T_conv_ produce IL-2 upon activation and then gradually upregulate CD25. Since T_reg_ constitutively express CD25, they can sense and signal via IL-2 as soon as it is produced, assuming that these T_reg_ are within suitable proximity to the cytokine secreting cells. Because IL-2 signaling further upregulates CD25, T_reg_ can even increase their ability to capture IL-2 as compared to T_conv_ which need to initiate CD25 expression post TCR-mediated activation (Feinerman et al., 2010). Indeed, it has been shown *in vivo* that T_reg_ are the first cells to respond to IL-2 upon antigenic challenge of T_conv_ (O’Gorman et al., 2009). As the amount of IL-2 produced by T-cells correlates with the extent of co-stimulation from DC *in vitro* (Shahinian et al., 1993) and *in vivo* (Kastenmuller et al., 2011), it might reflect the magnitude of pathogen burden and the extent of innate stimulation. Therefore, T_reg_ “sense” the initiation of an adaptive immune response in a qualitative and potentially quantitative manner when responding to IL-2 signals derived from adaptive effectors.

## T_reg_ Control the Availability of IL-2

Given the relative abundance of T_reg_ in SLO, where adaptive responses are initiated and IL-2 is being produced, it seems likely that the “sensing” of IL-2 by T_reg_ consumes a significant amount of the totally available IL-2. In this scenario, the mere presence of T_reg_ could reduce IL-2 availability and limit T_eff_ responses, without a need for active regulation (cytokine-sink model). Indeed, the presence of T_reg_ leads to substantial competition for IL-2, resulting in impaired proliferation of T_eff_ cells *in vitro*. Competition was further demonstrated *in vivo*, with a primary effect on the survival of T_eff_ and not on their proliferation (Pandiyan et al., 2007; Kastenmuller et al., 2011). T_reg_ also control IL-2 production (Thornton and Shevach, 1998), either by directly acting on T-cells (Bodor et al., 2007; Vaeth et al., 2011) or indirectly, through DC (Onishi et al., 2008). The latter concept is based on *in vitro* evidence of a positive correlation between IL-2 production by Teff, the strength of ConA stimulation, and the amount of CD28 expression (Shahinian et al., 1993). T_reg_ express significant levels of surface CTLA-4 on their surface and this molecule can directly block co-stimulatory molecules and CD28-CD80/86-interactions, or, via trans endocytosis, modulate the amount of CD80/86 that is displayed by DC (Wing et al., 2008; Qureshi et al., 2011). Importantly, the amount of CTLA-4 expressed on T_reg_ is again regulated by IL-2 signals. Consequently, T_reg_ control the level of co-stimulation through CD80/CD86 surface expression not only during steady-state (Schildknecht et al., 2010), but, importantly, also during highly inflammatory processes such as viral infection (Kastenmuller et al., 2011).

**Figure 1:**
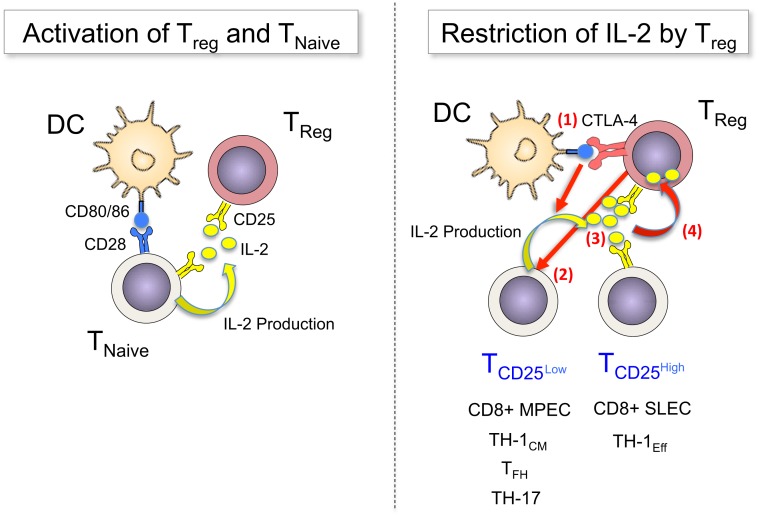
**T_reg_ sense IL-2 early during immune responses and restrict cytokine availability to control T-cell fates**. *Left panel:* TCR signals (not depicted) and co-stimulatory signals via CD80/86 activate naïve T-cells to upregulate CD25 and produce IL-2. T_reg_ have constitutive expression of CD25, which enables them to “sense” early (d0) production of IL-2 and become activated. *Right panel:* Once activated T-cells divide, they become heterogeneous for CD25 expression (d2–4): a fraction of cells downregulates CD25, avoiding further IL-2 signals – and potentially regulation through T_reg_ – in order to differentiate into long-lived memory cells (MPEC or TH-1_CM_), follicular helper cells or TH-17 cells, or they maintain CD25 expression for prolonged IL-2 signals to undergo terminal effector differentiation. T_reg_ can balance this differentiation processes by regulating the availability of IL-2 via multiple mechanisms, among them (1) the blocking and reduction of CD80/86 via CTLA-4, (2) the direct inhibition of IL-2 production by T-cells, (3) the competition for IL-2, and (4) the consumption and degradation of IL-2.

Therefore, DC appear to constitute a platform on which both stimulation and regulation of conventional T-cells is executed, with IL-2 being a central mediator that activates both T_eff_ and T_reg_. Feed-back loops involving the constitutive high levels of CD25 on T_regs_ and the IL-2-promoted upregulation of CTLA-4 on these cells operate in concert to restrict IL-2 availability to activated T_eff_, controlling their expansion, differentiation, and survival (Figure 1).

## T_reg_ Selectively Regulate Effector T-Cell Responses but Preserve Memory Development

Recently, the effects of IL-2 during acute infection have been further refined through analysis of the role of this cytokine in the various stages of CD8+ T-cell differentiation. After their initial activation and induction of CD25 expression, a subpopulation of CD8+ T-cells decrease CD25 levels and become unresponsive to further IL-2 signals, and, at the same time, upregulate the IL-7Rα-chain and develop into long-lived memory cells. This is in contrast to T-cells that are exposed to IL-2 for a prolonged period and maintain CD25 expression, undergo enhanced expansion, but differentiate into short-lived effector cells (SLEC) that are prone to apoptosis and severe population contraction after the peak of the response (Kalia et al., 2010; Obar et al., 2010; Pipkin et al., 2010). The recent development of genetic models allowing the specific depletion of Foxp3^+^ T_reg_ (Kim et al., 2007; Lahl et al., 2007) without blocking IL-2/CD25 interactions (Murakami et al., 2002; Suvas et al., 2003; Toka et al., 2004; Heit et al., 2008) enabled asking whether T_reg_ differentially affect these CD8+ T-cell subpopulations. Indeed, manipulating the numbers of activated T_reg_ *in vivo* impacted CD25 expression on activated CD8+ T-cells, indicative of altered IL-2 availability. This resulted in specific changes in numbers of SLEC while leaving the memory CD8+ T-cell compartment largely unaltered (Kastenmuller et al., 2011; McNally et al., 2011). Interestingly, the “window of opportunity” for the regulation of CD8+ T-cell responses by T_reg_ overlapped with the time of CD25 expression on CD8+ T-cells: depletion of T_reg_ cells as late as d2–3 post infection enhanced antigen-specific T-cell numbers in a viral infection model, but depletion later than d5 (when CD8+ T-cells do not express CD25) failed to do so (Kastenmuller et al., 2011). In addition, enhanced expansion of SLEC in the absence of T_reg_ was abrogated through the neutralization of IL-2 (McNally et al., 2011). Therefore, by controlling the availability of IL-2, T_reg_ cells can act as rheostats that balance the differentiation and expansion of pathogen-specific CD8+ effector T-cells. Importantly, by selectively regulating SLEC over memory precursor T-cells, which rapidly seem to become independent of IL-2 signals (d2–3), T_reg_ can limit the extent of acute effector responses without blunting the development of long-lived memory (Kastenmuller et al., 2011). However, once memory is formed and the host is re-challenged with a pathogen, T_reg_ can again control the expansion of secondary effector cells adapted to the extent and requirements of the current infection.

## T_reg_ Mediated Restriction of IL-2 as a General Mechanism to Regulate Fate Decisions in T-Cells

Beyond CD8+ T-cells, IL-2 likely serves as a central element that allows T_reg_ to regulate population size and differentiation of T-cells in general. A series of recent studies have established the role of IL-2 in CD4+ T-cell differentiation into T_H1_, T_H2_, T_H17_, and T_FH_ cells (Liao et al., 2011; Boyman and Sprent, 2012). As with CD8+ T-cells discussed above, CD4+ T-cells segregate into CD25^high^ and CD25^low^ cells within the first days of a response to an acute infection, and prolonged IL-2 signals in CD25^high^ cells leads to terminal differentiation and Blimp1 and T-bet upregulation in CD4+ effector cells. In contrast, CD25^low^ cells gave rise to long-lived CXCR5^high^CCR7^high^T-bet^low^ precursors of central memory cells, as well as CXCR5^high^Bcl6^high^ follicular T-helper cells (T_FH_; Choi et al., 2011; Pepper et al., 2011). In line with this, T_reg_ limit the expansion of antiviral CD4+ during acute infection, yet do not influence the generation of neutralizing antibodies (Kastenmuller et al., 2011).

Therefore, by limiting excessive IL-2, T_reg_ might not only blunt T_eff_ response but also ensure the generation of T_FH_ cells and consequently the development of appropriate humoral immunity early during acute infection, as IL-2 signals negatively regulate T_FH_ differentiation (Ballesteros-Tato et al., 2012; Johnston et al., 2012). By restricting IL-2 during acute infection, T_reg_ might additionally enhance mucosal immunity and regeneration (potentially preventing superinfection) through promotion of T_H17_ generation, because production of this class of effector cells is inhibited by IL-2 (Chen et al., 2011; Pandiyan et al., 2011).

## Summary and Perspective

In summary, we propose a model in which IL-2 availability is a central factor that controls the magnitude and shapes the character of adaptive immune responses. T_reg_ control access of other T-cells to this crucial cytokine by limiting its production through interference with co-stimulatory molecule availability on DC, as well as by reducing its abundance through consumption. Importantly, this does not act to simply blunt the overall immune response but selectively impacts on T-cell fates that require larger amounts of IL-2. In contrast, other T-cell subpopulations, such as memory-precursors or T_FH_, are not suppressed, allowing for the generation of cellular and humoral immunological memory to protect the host from future pathogen encounters (Figure 1). T_reg_ are therefore not merely immunosuppressive, they actively participate in guiding the differentiation and fate decisions of other T-cells by regulating the availability of IL-2 in SLO. In this regard, T_reg_ controlling IL-2 availability remind us of the three Moirai (the incarnation of destiny in greek mythology) who controlled the thread of life and thereby directed the fate of individuals.

In addition to this early regulation of effector responses through IL-2, T_reg_ can undergo functional specialization that parallels the differentiation of conventional CD4+ T-cells in terms of transcription factor usage and expression of chemokine receptors important for homing to peripheral sites (Chaudhry et al., 2009; Koch et al., 2009; Zheng et al., 2009; Chung et al., 2011; Linterman et al., 2011). This differentiation might facilitate T_reg_ control of fully differentiated effector cells in infected tissues, which is likely to involve mechanisms distinct from regulating or competing for IL-2 (Soper et al., 2007), such as the production of immunosuppressive cytokines (Rubtsov et al., 2008) or cytotoxic molecules (Cao et al., 2007; Loebbermann et al., 2012).

Based on the emerging picture of selective control of effector T-cell fates, we speculate that interfering with T_reg_ function will help to optimize short-term immunotherapeutic approaches, but might be less promising to increase the efficacy of prophylactic vaccines aiming at the induction of long-term memory through T- and B-cells.

## References

[B1] Ballesteros-Tato A. Leon B. Graf B. A. Moquin A. Adams P. S. Lund F. E. Randall T. D. (2012). Interleukin-2 inhibits germinal center formation by limiting T follicular helper cell differentiation. Immunity 36, 847–856 10.1016/j.immuni.2012.02.012 22464171 PMC3361521

[B2] Belkaid Y. Tarbell K. (2009). Regulatory T cells in the control of host-microorganism interactions (*). Annu. Rev. Immunol. 27, 551–589 10.1146/annurev.immunol.021908.132723 19302048

[B3] Bodor J. Fehervari Z. Diamond B. Sakaguchi S. (2007). ICER/CREM-mediated transcriptional attenuation of IL-2 and its role in suppression by regulatory T cells. Eur. J. Immunol. 37, 884–895 10.1002/eji.200636510 17372992

[B4] Boyman O. Sprent J. (2012). The role of interleukin-2 during homeostasis and activation of the immune system. Nat. Rev. Immunol. 12, 180–190 22343569 10.1038/nri3156

[B5] Cao X. Cai S. F. Fehniger T. A. Song J. Collins L. I. Piwnica-Worms D. R. Ley T. J. (2007). Granzyme B and perforin are important for regulatory T cell-mediated suppression of tumor clearance. Immunity 27, 635–646 10.1016/j.immuni.2007.08.014 17919943

[B6] Chaudhry A. Rudra D. Treuting P. Samstein R. M. Liang Y. Kas A. Rudensky A. Y. (2009). CD4+ regulatory T cells control TH17 responses in a Stat3-dependent manner. Science 326, 986–991 10.1126/science.1172702 19797626 PMC4408196

[B7] Chen Y. Haines C. J. Gutcher I. Hochweller K. Blumenschein W. M. McClanahan T. Hammerling G. Li M. O. Cua D. J. McGeachy M. J. (2011). Foxp3(+) regulatory T cells promote T helper 17 cell development in vivo through regulation of interleukin-2. Immunity 34, 409–421 10.1016/j.immuni.2011.02.011 21435588

[B8] Choi Y. S. Kageyama R. Eto D. Escobar T. C. Johnston R. J. Monticelli L. Lao C. Crotty S. (2011). ICOS receptor instructs T follicular helper cell versus effector cell differentiation via induction of the transcriptional repressor Bcl6. Immunity 34, 932–946 10.1016/j.immuni.2011.03.023 21636296 PMC3124577

[B9] Chung Y. Tanaka S. Chu F. Nurieva R. I. Martinez G. J. Rawal S. Wang Y. H. Lim H. Reynolds J. M. Zhou X. H. Fan H. M. Liu Z. M. Neelapu S. S. Dong C. (2011). Follicular regulatory T cells expressing Foxp3 and Bcl-6 suppress germinal center reactions. Nat. Med. 17, 983–988 10.1038/nm.2426 21785430 PMC3151340

[B10] Feinerman O. Jentsch G. Tkach K. E. Coward J. W. Hathorn M. M. Sneddon M. W. Emonet T. Smith K. A. Altan-Bonnet G. (2010). Single-cell quantification of IL-2 response by effector and regulatory T cells reveals critical plasticity in immune response. Mol. Syst. Biol. 6, 437 10.1038/msb.2010.90 21119631 PMC3010113

[B11] Fontenot J. D. Rasmussen J. P. Gavin M. A. Rudensky A. Y. (2005). A function for interleukin 2 in Foxp3-expressing regulatory T cells. Nat. Immunol. 6, 1142–1151 10.1038/ni1179 16227984

[B12] Heit A. Gebhardt F. Lahl K. Neuenhahn M. Schmitz F. Anderl F. Wagner H. Sparwasser T. Busch D. H. Kastenmuller K. (2008). Circumvention of regulatory CD4(+) T cell activity during cross-priming strongly enhances T cell-mediated immunity. Eur. J. Immunol. 38, 1585–1597 10.1002/eji.200737966 18465771

[B13] Johnston R. J. Choi Y. S. Diamond J. A. Yang J. A. Crotty S. (2012). STAT5 is a potent negative regulator of TFH cell differentiation. J. Exp. Med. 209, 243–250 10.1084/jem.20111174 22271576 PMC3281266

[B14] Josefowicz S. Z. Lu L. F. Rudensky A. Y. (2012). Regulatory T cells: mechanisms of differentiation and function. Annu. Rev. Immunol. 30, 531–564 10.1146/annurev.immunol.25.022106.141623 22224781 PMC6066374

[B15] Kalia V. Sarkar S. Subramaniam S. Haining W. N. Smith K. A. Ahmed R. (2010). Prolonged interleukin-2R alpha expression on virus-specific CD8+ T cells favors terminal-effector differentiation in vivo. Immunity 32, 91–103 10.1016/j.immuni.2009.11.010 20096608

[B16] Kastenmuller W. Gasteiger G. Subramanian N. Sparwasser T. Busch D. H. Belkaid Y. Drexler I. Germain R. N. (2011). Regulatory T cells selectively control CD8+ T cell effector pool size via IL-2 restriction. J. Immunol. 187, 3186–3197 10.4049/jimmunol.1101649 21849683 PMC3169715

[B17] Kim J. M. Rasmussen J. P. Rudensky A. Y. (2007). Regulatory T cells prevent catastrophic autoimmunity throughout the lifespan of mice. Nat. Immunol. 8, 191–197 10.1038/ni1428 17136045

[B18] Koch M. A. Tucker-Heard G. Perdue N. R. Killebrew J. R. Urdahl K. B. Campbell D. J. (2009). The transcription factor T-bet controls regulatory T cell homeostasis and function during type 1 inflammation. Nat. Immunol. 10, 595–602 10.1038/nrg2630 19412181 PMC2712126

[B19] Lahl K. Loddenkemper C. Drouin C. Freyer J. Arnason J. Eberl G. Hamann A. Wagner H. Huehn J. Sparwasser T. (2007). Selective depletion of Foxp3+ regulatory T cells induces a scurfy-like disease. J. Exp. Med. 204, 57–63 10.1084/jem.20061852 17200412 PMC2118432

[B20] Liao W. Lin J. X. Leonard W. J. (2011). IL-2 family cytokines: new insights into the complex roles of IL-2 as a broad regulator of T helper cell differentiation. Curr. Opin. Immunol. 23, 598–604 10.1016/j.coi.2011.08.003 21889323 PMC3405730

[B21] Linterman M. A. Pierson W. Lee S. K. Kallies A. Kawamoto S. Rayner T. F. Srivastava M. Divekar D. P. Beaton L. Hogan J. J. Fagarasan S. Liston A. Smith K. G. Vinuesa C. G. (2011). Foxp3+ follicular regulatory T cells control the germinal center response. Nat. Med. 17, 975–982 10.1038/nm.2425 21785433 PMC3182542

[B22] Loebbermann J. Thornton H. Durant L. Sparwasser T. Webster K. E. Sprent J. Culley F. J. Johansson C. Openshaw P. J. (2012). Regulatory T cells expressing granzyme B play a critical role in controlling lung inflammation during acute viral infection. Mucosal Immunol. 5, 161–172 10.1038/mi.2011.62 22236998 PMC3282434

[B23] Malek T. R. Castro I. (2010). Interleukin-2 receptor signaling: at the interface between tolerance and immunity. Immunity 33, 153–165 10.1016/j.immuni.2010.08.004 20732639 PMC2946796

[B24] McNally A. Hill G. R. Sparwasser T. Thomas R. Steptoe R. J. (2011). CD4+ CD25+ regulatory T cells control CD8+ T-cell effector differentiation by modulating IL-2 homeostasis. Proc. Natl. Acad. Sci. U.S.A. 108, 7529–7534 10.1073/pnas.1101825108 21502514 PMC3088596

[B25] Murakami M. Sakamoto A. Bender J. Kappler J. Marrack P. (2002). CD25+CD4+ T cells contribute to the control of memory CD8+ T cells. Proc. Natl. Acad. Sci. U. S. A. 99, 8832–8837 10.1073/pnas.132254399 12084927 PMC124384

[B26] Obar J. J. Molloy M. J. Jellison E. R. Stoklasek T. A. Zhang W. Usherwood E. J. Lefrancois L. (2010). CD4+ T cell regulation of CD25 expression controls development of short-lived effector CD8+ T cells in primary and secondary responses. Proc. Natl. Acad. Sci. U.S.A. 107, 193–198 10.1073/pnas.0909945107 19966302 PMC2806751

[B27] O’Gorman W. E. Dooms H. Thorne S. H. Kuswanto W. F. Simonds E. F. Krutzik P. O. Nolan G. P. Abbas A. K. (2009). The initial phase of an immune response functions to activate regulatory T cells. J. Immunol. 183, 332–339 10.4049/jimmunol.0900691 19542444 PMC2753472

[B28] Onishi Y. Fehervari Z. Yamaguchi T. Sakaguchi S. (2008). Foxp3+ natural regulatory T cells preferentially form aggregates on dendritic cells in vitro and actively inhibit their maturation. Proc. Natl. Acad. Sci. U.S.A. 105, 10113–10118 10.1073/pnas.0711106105 18635688 PMC2481354

[B29] Pandiyan P. Conti H. R. Zheng L. Peterson A. C. Mathern D. R. Hernandez-Santos N. Edgerton M. Gaffen S. L. Lenardo M. J. (2011). CD4(+)CD25(+) Foxp3(+) regulatory T cells promote Th17 cells in vitro and enhance host resistance in mouse Candida albicans Th17 cell infection model. Immunity 34, 422–434 10.1016/j.immuni.2011.03.002 21435589 PMC3258585

[B30] Pandiyan P. Zheng L. Ishihara S. Reed J. Lenardo M. J. (2007). CD4+ CD25+ Foxp3+ regulatory T cells induce cytokine deprivation-mediated apoptosis of effector CD4+ T cells. Nat. Immunol. 8, 1353–1362 10.1038/ni1536 17982458

[B31] Pepper M. Pagan A. J. Igyarto B. Z. Taylor J. J. Jenkins M. K. (2011). Opposing signals from the Bcl6 transcription factor and the interleukin-2 receptor generate T helper 1 central and effector memory cells. Immunity 35, 583–595 10.1016/j.immuni.2011.09.009 22018468 PMC3208313

[B32] Pipkin M. E. Sacks J. A. Cruz-Guilloty F. Lichtenheld M. G. Bevan M. J. Rao A. (2010). Interleukin-2 and inflammation induce distinct transcriptional programs that promote the differentiation of effector cytolytic T cells. Immunity 32, 79–90 10.1016/j.immuni.2009.11.012 20096607 PMC2906224

[B33] Qureshi O. S. Zheng Y. Nakamura K. Attridge K. Manzotti C. Schmidt E. M. Baker J. Jeffery L. E. Kaur S. Briggs Z. Hou T. Z. Futter C. E. Anderson G. Walker L. S. Sansom D. M. (2011). Trans-endocytosis of CD80 and CD86: a molecular basis for the cell-extrinsic function of CTLA-4. Science 332, 600–603 10.1126/science.1202947 21474713 PMC3198051

[B34] Rubtsov Y. P. Rasmussen J. P. Chi E. Y. Fontenot J. Castelli L. Ye X. Treuting P. Siewe L. Roers A. Henderson W. R. Jr. Muller W. Rudensky A. Y. (2008). Regulatory T cell-derived interleukin-10 limits inflammation at environmental interfaces. Immunity 28, 546–558 10.1016/j.immuni.2008.02.017 18387831

[B35] Sadlack B. Merz H. Schorle H. Schimpl A. Feller A. C. Horak I. (1993). Ulcerative colitis-like disease in mice with a disrupted interleukin-2 gene. Cell 75, 253–261 10.1016/0092-8674(93)80067-O 8402910

[B36] Sakaguchi S. Sakaguchi N. Asano M. Itoh M. Toda M. (1995). Immunologic self-tolerance maintained by activated T cells expressing IL-2 receptor alpha-chains (CD25). Breakdown of a single mechanism of self-tolerance causes various autoimmune diseases. J. Immunol. 155, 1151–1164 7636184

[B37] Schildknecht A. Brauer S. Brenner C. Lahl K. Schild H. Sparwasser T. Probst H. C. Van Den Broek M. (2010). FoxP3+ regulatory T cells essentially contribute to peripheral CD8+ T-cell tolerance induced by steady-state dendritic cells. Proc. Natl. Acad. Sci. U.S.A. 107, 199–203 10.1073/pnas.0910620107 20018763 PMC2806715

[B38] Shahinian A. Pfeffer K. Lee K. P. Kundig T. M. Kishihara K. Wakeham A. Kawai K. Ohashi P. S. Thompson C. B. Mak T. W. (1993). Differential T cell costimulatory requirements in CD28-deficient mice. Science 261, 609–612 10.1126/science.7688139 7688139

[B39] Shevach E. M. (2009). Mechanisms of foxp3+ T regulatory cell-mediated suppression. Immunity 30, 636–645 10.1016/j.immuni.2009.04.010 19464986

[B40] Smith K. A. (2006). The structure of IL2 bound to the three chains of the IL2 receptor and how signaling occurs. Med. Immunol. 5, 3 10.1186/1476-9433-5-3 16907989 PMC1562422

[B41] Soper D. M. Kasprowicz D. J. Ziegler S. F. (2007). IL-2Rbeta links IL-2R signaling with Foxp3 expression. Eur. J. Immunol. 37, 1817–1826 10.1002/eji.200737101 17559173

[B42] Suvas S. Kumaraguru U. Pack C. D. Lee S. Rouse B. T. (2003). CD4+CD25+ T cells regulate virus-specific primary and memory CD8+ T cell responses. J. Exp. Med. 198, 889–901 10.1084/jem.20030171 12975455 PMC2194203

[B43] Suzuki H. Kundig T. M. Furlonger C. Wakeham A. Timms E. Matsuyama T. Schmits R. Simard J. J. Ohashi P. S. Griesser H. Taniguchi T. Paige C. J. Mak T. W. (1995). Deregulated T cell activation and autoimmunity in mice lacking interleukin-2 receptor beta. Science 268, 1472–1476 10.1126/science.7770771 7770771

[B44] Thornton A. M. Shevach E. M. (1998). CD4+ CD25+ immunoregulatory T cells suppress polyclonal T cell activation in vitro by inhibiting interleukin 2 production. J. Exp. Med. 188, 287–296 10.1084/jem.188.2.287 9670041 PMC2212461

[B45] Toka F. N. Suvas S. Rouse B. T. (2004). CD4+ CD25+ T cells regulate vaccine-generated primary and memory CD8+ T-cell responses against herpes simplex virus type 1. J. Virol. 78, 13082–13089 10.1128/JVI.78.23.13082-13089.2004 15542660 PMC525021

[B46] Vaeth M. Gogishvili T. Bopp T. Klein M. Berberich-Siebelt F. Gattenloehner S. Avots A. Sparwasser T. Grebe N. Schmitt E. Hunig T. Serfling E. Bodor J. (2011). Regulatory T cells facilitate the nuclear accumulation of inducible cAMP early repressor (ICER) and suppress nuclear factor of activated T cell c1 (NFATc1). Proc. Natl. Acad. Sci. U.S.A. 108, 2480–2485 10.1073/pnas.1009463108 21262800 PMC3038697

[B47] Willerford D. M. Chen J. Ferry J. A. Davidson L. Ma A. Alt F. W. (1995). Interleukin-2 receptor alpha chain regulates the size and content of the peripheral lymphoid compartment. Immunity 3, 521–530 10.1016/1074-7613(95)90180-9 7584142

[B48] Wing K. Onishi Y. Prieto-Martin P. Yamaguchi T. Miyara M. Fehervari Z. Nomura T. Sakaguchi S. (2008). CTLA-4 control over Foxp3+ regulatory T cell function. Science 322, 271–275 10.1126/science.1160062 18845758

[B49] Zheng Y. Chaudhry A. Kas A. Deroos P. Kim J. M. CHU T. T. Corcoran L. Treuting P. Klein U. Rudensky A. Y. (2009). Regulatory T-cell suppressor program co-opts transcription factor IRF4 to control T(H)2 responses. Nature 458, 351–356 10.1038/nature07856 19182775 PMC2864791

